# Spatial early-warning assessment of ecological security in the Poyang Lake Basin based on PSR and Spatial Durbin Modeling

**DOI:** 10.1038/s41598-026-44479-4

**Published:** 2026-04-24

**Authors:** HaiXiang Guo, CongLe Hu, MeiYi Xin, Qi Wu

**Affiliations:** 1 School of Economics and Management, China University of Geosciences, Wuhan, 430074 China; 2 The Laboratory of Natural Disaster Risk Prevention and Emergency Management of China University of Geosciences, Wuhan, 430074 China

**Keywords:** Ecological security, Early-warning, PSR model, SDM model, Ecology, Ecology, Environmental sciences, Environmental social sciences

## Abstract

Constructing a basin-level ecological security early-warning (ESEW) system is critical for overcoming the fragmentation of administrative management and achieving holistic watershed governance. Focusing on the Poyang Lake Basin, this study develops a spatial ESEW assessment framework based on the Pressure-State-Response (PSR) model. By integrating 1 km grid-scale data with the Spatial Durbin Model (SDM), we investigate the spatiotemporal evolution of ecological security and quantify the cross-regional spillover effects of key drivers from 2000 to 2020. The results reveal a structural “deterioration-then-recovery” trajectory: the area of “Moderate Warning” expanded from 52.40% in 2000 to a peak of 64.53% in 2010 due to rapid industrialization, before dropping to 37.93% in 2020 as “Light Warning” areas became dominant (62.00%). Spatial analysis confirms significant clustering of ecological risks. Crucially, the SDM identifies a strong spatial spillover effect, indicating that ecological degradation is highly contagious across county borders. Specifically, industrial sulfur dioxide emissions and urbanization intensity (NUADI) in neighboring areas exert significant negative spillovers on local ecological security, whereas farmland-to-forest conversion generates positive externalities. Based on these findings, we propose a differentiated governance strategy, including “Source Control” for high-risk pollution clusters and market-oriented ecological compensation mechanisms to internalize the benefits of upstream protection, providing a scientific basis for cross-regional collaborative governance.

**Supplementary Information:**

The online version contains supplementary material available at 10.1038/s41598-026-44479-4.

## Introduction

Against the backdrop of accelerating climate change and biodiversity loss, ecological security has become central to sustainable development^1^. River basins integrate upstream and downstream habitats with socioeconomic systems, shaping water supply, ecological regulation, and human–environment interactions^2^. A basin-scale ecological early-warning mechanism can support more anticipatory governance by diagnosing spatial disparities in ecosystem functioning and environmental quality, thereby enabling a shift from reactive restoration to targeted, evidence-based management.

Poyang Lake, the largest river-connected lake in the middle reaches of the Yangtze River, is pivotal for maintaining hydrological connectivity and regulating basin water resources. It also provides wintering habitat for 98% of the world’s Siberian cranes and serves as a key refuge for the Yangtze finless porpoise^3^. Yet intensifying human activities and climate change have increased environmental stress, contributing to wetland degradation and broader water and environmental pressures^4^. The Poyang Lake Basin is a coupled social–ecological–economic system in which ecological risks propagate along upstream, midstream, and downstream pathways; conditions in headwaters and midstream corridors directly influence the security of downstream lake wetlands. Because watershed boundaries do not align neatly with administrative jurisdictions—and the basin extends into neighboring provinces such as Fujian and Zhejiang—governance fragmentation can undermine basin-wide integrity. Accordingly, this study examines the temporal evolution of ecological security in the Poyang Lake Basin and provides evidence to support cross-regional collaborative governance.

Ecological safety early warning is commonly operationalized as an advance alert mechanism that identifies when an ecosystem is approaching or has exceeded critical thresholds. Two complementary strands underpin current early-warning research. First, resilience-based studies emphasize spatial early-warning signals of regime shifts, where increasing spatial autocorrelation or changes in patch-size distributions can foreshadow loss of resilience^5^. Second, assessment-oriented studies translate complex socio-ecological pressures and states into composite indices to support decision-making in heterogeneous systems. Such applications span cropland^6^, lakes^7^, cities^8^, river basins^9,10^, rivers^11^, and soils^12^. Methodologically, researchers have adopted the composite index method^13^, the Pressure–State–Response (PSR) model^14^, system dynamics (SD)^15^, back-propagation neural networks^16^, TOPSIS^17,18^, and the DPSIRM model^19^. Empirical studies further demonstrate the value of capturing nonlinear responses and hysteresis in lake systems^7^ and combining monitoring–remote-sensing data for spatiotemporal simulations of soil environmental capacity^12^. From a scale perspective, early-warning assessments have been implemented at provincial^20,21^, municipal^22,23^, and county scales^24^, and PSR-based implementations have been continuously refined for ecological contexts^25^. Importantly, some studies have begun to bridge assessment and governance mechanisms by introducing spatial econometric tools (e.g., SDM) to examine governance drivers such as public participation^26^.

Despite these advances, the literature shows two persistent gaps. First, many “early-warning” applications remain methodologically close to ecological security status assessment, and the spatial transmission mechanism of ecological risks across administrative boundaries is often discussed qualitatively rather than quantified. Second, although a growing body of work documents spatial dependence in ecological phenomena—such as sensitivity-based identification of local drivers of ecological vulnerability^27^, spatial autocorrelation of water ecological security^28^, and the spatial association between soil types and ecosystem service values^29^—existing studies usually focus either on spatial clustering of the overall status or on a single-factor relationship, leaving the synergistic interaction among multiple drivers insufficiently examined and their relative contributions unquantified.

Recent econometric studies, particularly those employing the Spatial Durbin Model, have improved causal inference on cross-regional effects by decomposing direct and indirect impacts of drivers such as new-type urbanization and public participation^26,30,31^. However, these SDM applications are rarely coupled with a high-resolution, grid-based early-warning index framework at the basin scale, which limits the ability to link fine-grained ecological risk diagnosis to cross-county spillover governance. Moreover, while PSR/DPSIR-style frameworks remain mainstream for constructing ecological security warning indices^32^, no unified, widely accepted evaluation framework has yet emerged for complex watershed systems. The PSR model proposed by the OECD in 1993 provides a structured logic for integrating pressure, state, and response, and has been used in different contexts such as island ecological risk assessment^33^ and nearshore eutrophication evaluation with explicit spatial heterogeneity^34^. Consistent with the “slow-variable” nature of many ecological processes, many studies therefore adopt 5–10 year observation intervals to capture structural change while avoiding noise-driven misinterpretation.

In summary, existing studies have developed mature methodological toolkits for constructing ecological security indices and for detecting spatial dependence. However, the field still lacks a clearly specified workflow that links multi-dimensional early-warning diagnosis to mechanism-consistent evidence on spillovers at policy-relevant administrative units. This limitation is particularly consequential in river-connected lake basins, where hydrological connectivity intensifies cross-jurisdictional interactions and increases the need for coordinated governance.

This study uses the Poyang Lake Basin as a case study to address limitations in existing ecological security assessment frameworks, particularly insufficient spatial resolution and the inadequate treatment of external pressures and spillover effects from surrounding jurisdictions. We develop a 1-km grid-based ecological security evaluation system grounded in the PSR framework to characterize spatiotemporal heterogeneity and the basin’s evolving dynamics. Using data from 2000, 2010, and 2020, we map the spatial patterns and temporal changes in ecological security across the basin. We then conduct global and local spatial autocorrelation analyses to delineate high-risk zones and apply spatial econometric models to quantify key drivers, including influences originating in neighboring areas, thereby informing the refinement of early-warning indicators. Finally, we translate the empirical findings into targeted warning metrics and collaborative governance strategies that are scientifically grounded and operationally feasible for the Poyang Lake Basin and comparable systems. While ecological security assessment typically offers a largely static description of environmental conditions, early warning serves a different scientific purpose. Its goal is to detect latent risks by identifying locations where ecological pressure has reached a critical level, even when an overt system collapse has not yet occurred. In highly dynamic watershed systems such as the Poyang Lake Basin, risks can propagate along upstream–downstream pathways and accumulate in nonlinear ways, making early warning essential for capturing incipient degradation signals and avoiding irreversible transitions. In this study, “early warning” is defined operationally in a way that differs from conventional time-series forecasting. Many early-warning systems rely on high-frequency temporal data to predict the timing of future regime shifts; by contrast, we conceptualize ESEW as a state-based warning system. Here, a “warning” refers to identifying threshold exceedances and diagnosing spatially explicit high-risk zones where current ecological pressure surpasses system resilience. By classifying the system into discrete risk states, the framework provides an actionable alert for policymakers by prioritizing areas requiring timely intervention, rather than projecting a specific future trajectory.

This study does not introduce a new spatial econometric estimator. Instead, it advances ecological security early-warning research by integrating and adapting methods in a basin-oriented manner. First, we develop a multi-scale, grid-to-county coupled ESEW framework that implements state-based warning at 1-km grids and then aggregates results to the county level for spillover diagnosis, thereby connecting fine-resolution risk identification with cross-jurisdictional transmission analysis. Second, to better capture anthropogenic structural pressure at high spatial resolution, we propose the Net Urban–Agrarian Dominance Index (NUADI) and downscale emission-related pressures to 1-km grids using nighttime lights, mitigating the mismatch between yearbook-based statistics and pixel-level mapping. Third, to better reflect the physical transmission processes in a connected lake–river basin, we introduce and validate a hydrological-network-based spatial weight matrix alongside conventional distance- and contiguity-based matrices. This design enables spillover inference that is consistent with upstream–downstream pathways and provides a more mechanism-aligned basis for basin-wide collaborative governance.

## Materials and methodology

### Study area

The Poyang Lake Basin covers 162,225 km², accounting for approximately 9% of the Yangtze River Basin. Most of the basin (156,743 km²; 96.62%) lies within Jiangxi Province, which corresponds to about 94% of Jiangxi’s total land area. The remaining 5,482 km² (3.38%) extends into parts of Fujian, Zhejiang, Anhui, Hunan, and Guangdong. Topographically, the basin is characterized by higher elevations in the northwest and lower terrain toward the southeast.

**Fig. 1:**
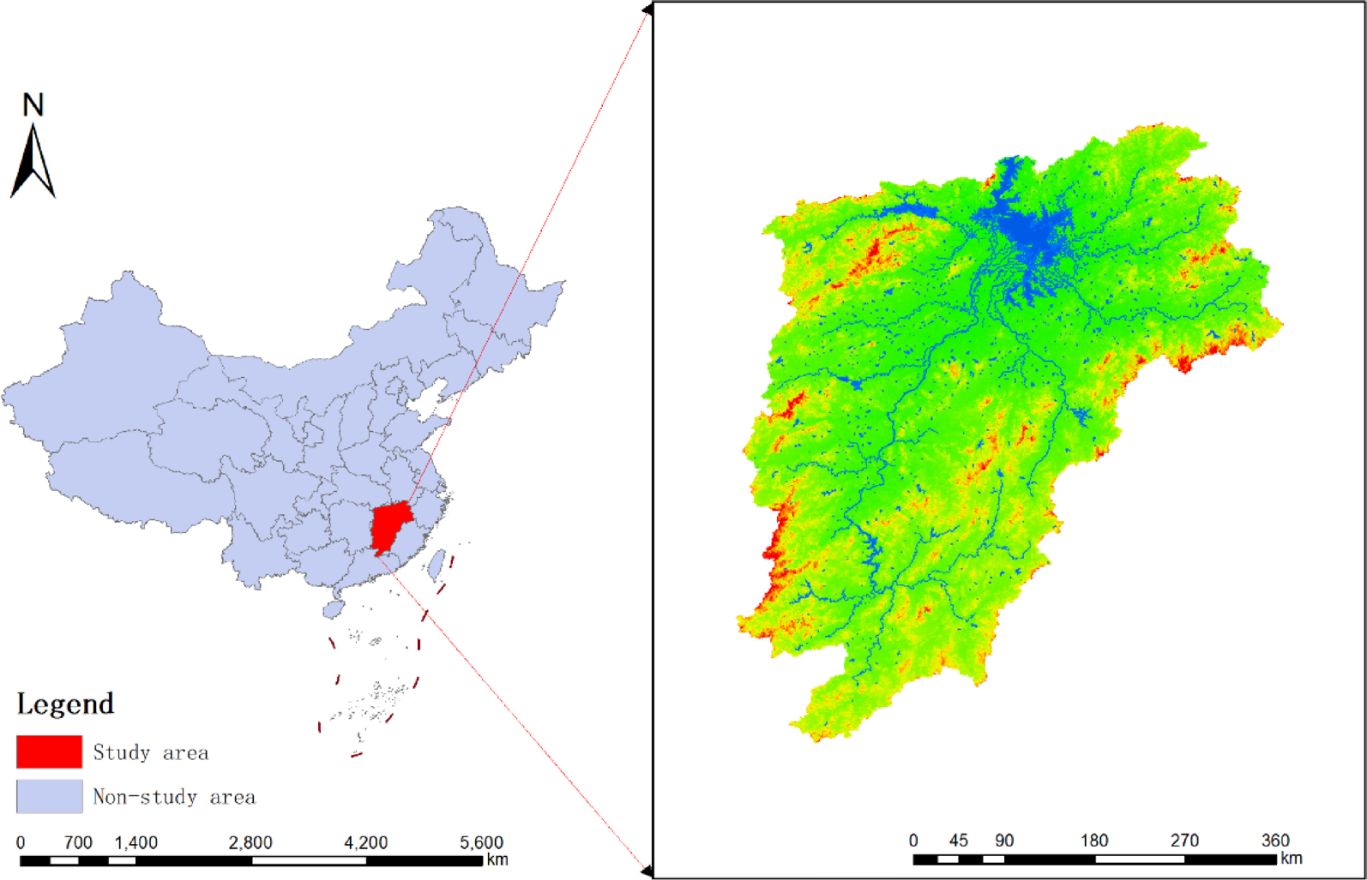
Location of the Poyang Lake Basin (The map was generated using ArcMap10.8 http://www.esri.com/).

In this study, we delineate the entire basin to reflect watershed integrity as a coupled natural–social–economic system. The basin exhibits a clear hierarchical organization: mountainous headwaters in the upper reaches function as water conservation areas, the middle reaches form a transition zone with intensive human activity, and the lower reaches converge into the lake–wetland system. Although the inter-provincial area represents only 3.38% of the basin, it includes critical headwater sections. Incorporating these segments allows us to explicitly examine how administrative boundaries may fragment governance and how ecological risks can propagate from upstream to downstream across provincial borders. The study area is shown in Fig. 1.

### Data source and processing

Precipitation and evaporation data for the Poyang Lake Basin are obtained from the China Meteorological Elements Average Status Spatial Interpolation Dataset. Land-use data with a resolution of 30 m are sourced from the China Multi-Period Land Use Remote Sensing Monitoring Dataset (CNLUCC). GDP and population distribution data are derived from the China GDP Spatial Distribution Kilometer-Grid Dataset and the China Population Spatial Distribution Kilometer-Grid Dataset, respectively. NDVI data are taken from the China Annual NDVI and EVI (1 km) Dataset. All of these datasets are available through the Resource and Environmental Science Data platform (http://www.resdc.cn/). Elevation data are collected from the Geospatial Data Cloud (https://www.gscloud.cn/). Indices such as the Net Urban-Agrarian Dominance Index, Biological Richness, Rate of Farmland Return to Forest and Grass, and the Conversion Rate of Ecological Land to Non-Ecological Land are calculated based on land-use data. Slope data are derived from the elevation data, and road network density data are extracted from Gaode Maps and the OSM (OpenStreetMap) platform. The nighttime light data uses the Chinese long-term time-series artificial nighttime light dataset from the Resources and Environmental Science Data Platform. Other socioeconomic statistics, such as the output of the tertiary industry, are taken from the statistical yearbooks of the respective regions.

Given the heterogeneity in data types and sources, all raster layers were harmonized to a 1-km resolution for early-warning mapping and index construction. We then used zonal statistics to aggregate grid-level information to the county level for the spatial econometric analysis. This nested, multi-scale design links fine-resolution ecological diagnosis to policy-relevant spillover inference. Specifically, the ESEW index is first computed at the 1-km grid scale to capture within-county heterogeneity and to identify spatially explicit hotspots that can be masked by administrative averaging. Spillover effects, in contrast, are estimated at the county scale because counties are the principal units for environmental governance in the basin and because socioeconomic covariates and regulatory responses are consistently observed and interpretable at this level, allowing a comparable panel structure and a tractable spatial weight matrix. After deriving grid-level indicators and ESEW values, we aggregated them to counties to obtain county-level ESEW and corresponding explanatory indicators (or principal components). We recognize that upscaling may attenuate local extremes; therefore, grid-scale maps are retained for diagnosing within-county hotspots, whereas the county-scale SDM is used to quantify cross-jurisdictional spillovers. Together, the two scales provide complementary evidence connecting fine-scale risk identification with governance-oriented spillover mechanisms.

To ensure reproducibility, we followed a standardized processing pipeline. A 1 km × 1 km fishnet was created in ArcGIS 10.8 (Create Fishnet) and used as the spatial reference for all raster layers. All source rasters were resampled to the 1-km grid, using nearest-neighbor resampling for categorical layers (e.g., land use) and bilinear interpolation for continuous surfaces. Principal Component Analysis (PCA) was implemented in SPSSAU after Z-score standardization of the 15 indicators; components were retained under the eigenvalue-greater-than-one criterion and rotated using Varimax to improve interpretability. For the county-level spatial analysis, an inverse-distance spatial weight matrix was constructed in Stata 16.0 based on Euclidean distances between county centroid coordinates and row-standardized. The Spatial Durbin Model was then estimated in Stata using the xsmle command.

### Methods

#### Ecological security early-warning (ESEW) index system and evaluation model

Step 1: Selecting Evaluation Indexes and Constructing the Index System

The Pressure–State–Response (PSR) framework provides a structured approach for diagnosing environmental problems by linking anthropogenic drivers (Pressure), current ecological conditions (State), and societal or policy interventions (Response). In this study, the PSR model is used to organize the indicator system for the grid-based ESEW assessment and to ensure that early-warning signals can be interpreted in terms of both drivers and governance levers.

The Pressure layer captures human-induced stresses that increase ecological load or reduce ecological space. We represent pressure through three complementary dimensions: population pressure (e.g., population density or related measures), pollution emission pressure associated with industrial, agricultural, and domestic outputs (e.g., wastewater discharge, air pollutants, and fertilizer use), and anthropogenic disturbance pressure that reflects the intensity and spatial footprint of human intervention. In particular, disturbance indices derived from remote sensing and land-use data are used to characterize land-use conversion, infrastructure expansion, and landscape fragmentation, which are central mechanisms through which human activities alter habitat continuity and ecosystem functioning.

The State layer describes the prevailing ecological background and environmental conditions that mediate or reflect the impacts of pressure. We therefore select natural-condition indicators—such as precipitation, biodiversity-related metrics, and slope—to represent the basin’s ecological context under accumulated pressures and to capture spatial heterogeneity relevant to watershed processes.

The Response layer reflects actions and adjustments that can mitigate pressures or enhance ecological resilience. We include indicators related to industrial structure optimization (e.g., a higher share of the tertiary sector, which is generally associated with lower resource intensity and emissions) and ecological restoration efforts such as Grain-for-Green that improve ecosystem services including soil and water conservation and carbon sequestration. In addition, conversion of ecological land to non-ecological uses is treated as a negative response signal, as it indicates weakening constraints on ecological space and potential governance failure. Together, these PSR-organized indicators allow the ESEW framework to identify where pressures are high, states are vulnerable, and responses are insufficient—conditions under which early-warning hotspots are most likely to emerge.

This study selects 2000, 2010, and 2020 as observation nodes to reflect policy periodicity, major strategic shifts, and the slow-moving nature of ecological change. These years correspond to key milestones in China’s ecological governance. The year 2000 is used as the baseline, representing the early stage of the “Return of Grain to Lake” policy implemented after the major floods in 1998. The year 2010 is treated as a pivotal turning point: following the State Council’s approval of the Poyang Lake Ecological Economic Zone Plan in late 2009, it captures basin conditions at the point when regional conservation was elevated to a national strategy. The year 2020 marks the end of the 13th Five-Year Plan and coincides with the drafting phase of the Yangtze River Protection Law, thereby reflecting the cumulative outcomes of a decade of intensified governance. Although the observations are decadal, this design is well suited to identifying the U-shaped shift in ecological security: 2000–2010 reflects stress accumulation under rapid industrialization, whereas 2010–2020 reflects recovery under strengthened national policy interventions, supporting an interpretation of governance mechanisms initiated around the 2010 pivot.

Importantly, the selection of 2020 is not merely historical. Many ecological security indicators, including land-use structure, vegetation conditions, and topographic constraints, evolve as slow variables; decadal windows are therefore commonly used to capture structural transitions rather than short-term fluctuations. In addition, 2020 provides a policy-relevant endpoint that enables evaluation of accumulated governance effects and examination of persistent spatial spillover mechanisms. Finally, because the proposed early-warning framework is state-based rather than time-series forecasting oriented, it focuses on diagnosing spatial threshold exceedances and identifying high-risk zones for targeted intervention; the framework remains readily extensible as new data become available. The index system constructed from the 2000, 2010, and 2020 datasets is summarized in Table 1.

**Table 1:** Ecological ESEW index system.

Target layer	Criteria layer	Index layer	Attribute	Formula
ESEW	P	P1: Population density	-	
		P2: Per capita GDP	-	
		P3:Net Urban-Agrarian Dominance Index	-	$(\mathrm{C}\mathrm{o}\mathrm{n}\mathrm{s}\mathrm{t}\mathrm{r}\mathrm{u}\mathrm{c}\mathrm{t}\mathrm{i}\mathrm{o}\mathrm{n}\mathrm{l}\mathrm{a}\mathrm{n}\mathrm{d}\mathrm{a}\mathrm{r}\mathrm{e}\mathrm{a}-\mathrm{C}\mathrm{u}\mathrm{l}\mathrm{t}\mathrm{i}\mathrm{v}\mathrm{a}\mathrm{t}\mathrm{e}\mathrm{d}\mathrm{a}\mathrm{r}\mathrm{e}\mathrm{a})/\mathrm{T}\mathrm{o}\mathrm{t}\mathrm{a}\mathrm{l}\mathrm{a}\mathrm{r}\mathrm{e}\mathrm{a}$
		P4: Per 10,000 yuan of GDP Industrial wastewater discharge	-	
		P5: Per 10,000 yuan of GDP Industrial sulfur dioxide emissions	-	
	S	S1: Annual precipitation	+	
		S2: Elevation	+	
		S3: Biological richness	+	(0.35×Forest land×0.21×Grassland + 0.28 × Water area + 0.11×Cultivated land + 0.04× Construction Land + 0.01×bare ground) / Total area
		S4: Annual evaporation	-	
		S5: NDVI	+	
		S6: Slope	+	
		S7: Road network density index	-	
	R	R1: Proportion of tertiary industry to GDP	+	
		R2: Rate of returning farmland to forest and grass	+	Area of cultivated land converted to forest and grassland / initial cultivated land area
		R3: Conversion rate of ecological land to nonecological land	-	(Area of forest, grassland, and water bodies converted into farmland, built-up land, or bare land) / (Initial area of forest, grassland, and water bodies)

To characterize the structural intensity of anthropogenic pressure, we constructed the Net Urban-Agrarian Dominance Index (NUADI). Unlike traditional cumulative interference metrics, this index quantifies the net trade-off between ‘hard’ impervious surfaces and ‘soft’ agricultural matrices. The formula is calculated as:

$$(\mathrm{C}\mathrm{o}\mathrm{n}\mathrm{s}\mathrm{t}\mathrm{r}\mathrm{u}\mathrm{c}\mathrm{t}\mathrm{i}\mathrm{o}\mathrm{n}\mathrm{l}\mathrm{a}\mathrm{n}\mathrm{d}\mathrm{a}\mathrm{r}\mathrm{e}\mathrm{a}-\mathrm{C}\mathrm{u}\mathrm{l}\mathrm{t}\mathrm{i}\mathrm{v}\mathrm{a}\mathrm{t}\mathrm{e}\mathrm{d}\mathrm{a}\mathrm{r}\mathrm{e}\mathrm{a})/\mathrm{T}\mathrm{o}\mathrm{t}\mathrm{a}\mathrm{l}\mathrm{a}\mathrm{r}\mathrm{e}\mathrm{a}$$

Where Construction Land represents irreversible habitat sealing and Cultivated Land represents semi-natural permeable landscapes. This metric effectively captures the gradient of land-use intensity, distinguishing between regions undergoing critical urban hardening (positive values) and those maintaining a predominantly agrarian ecological buffer (negative values).

Additionally, wastewater and sulfur dioxide emission data are assigned to 1 km grid cells based on the nighttime light index.

$$\begin{gathered} {\mathrm{Wastewater~Discharge~in~a~Given~Grid~Cell}} \hfill \\ =\left( \begin{gathered} {\mathrm{Total~Wastewater~Discharge~of~the~County}} \hfill \\ \times {\mathrm{Nighttime~Light~Index~of~the~Grid~Cell~in~that~County}} \hfill \\ \end{gathered} \right)/{\mathrm{Total~Nighttime~Light~Index~of~the~County}} \hfill \\ \end{gathered}$$

Sulfur dioxide emissions follow a similar approach. It is important to clarify the ecological directionality of topographic indicators (Slope and Elevation) within the specific context of the Poyang Lake Basin. While steep slopes generally imply higher soil erosion risks in natural hazard assessments, in the context of comprehensive ecological security, topography serves as a critical natural barrier against anthropogenic disturbance. The low-lying flatlands in the basin center are the primary zones for intensive urbanization and agriculture, accumulating the highest ecological pressure. Conversely, the peripheral high-altitude and steep-slope regions (e.g., the Wuyi and Luoxiao Mountains) function as ecological shelters with minimal human interference and high forest coverage. Therefore, in this study, Elevation and Slope are conceptualized as positive indicators (+), serving as proxies for ‘remoteness from human stress’ and ‘habitat integrity.

Step 2: Grid representation of evaluation metrics

Most existing studies on ecological security early warning are focused on the municipal or county level, which makes it challenging to capture internal variations within the study area. To address this, this study uses ArcGIS software to create 1 km × 1 km grids, subdividing the research region into smaller units. This approach provides a more intuitive representation of changes within each grid unit across the study area.

Step 3: Calculate the ESEW index

Standardization and Zero-Value Handling:

To eliminate dimensional differences, the range normalization method was applied. Since the Entropy method involves logarithmic calculations (lnx), zero values in standardized data can lead to calculation errors. Therefore, a shift constant was added to the standardized values to ensure mathematical validity. The formulas are:

For positive indexes: ${r}_{ij}=\frac{{x}_{ij}-\mathrm{min}\left({x}_{j}\right)}{\mathrm{max}\left({x}_{j}\right)-\mathrm{min}\left({x}_{j}\right)}+0.0001$

For negative indexes: ${r}_{ij}=\frac{\mathrm{max}\left({x}_{j}\right)-{x}_{ij}}{\mathrm{max}\left({x}_{j}\right)-\mathrm{min}\left({x}_{j}\right)}+0.0001$

Hierarchical Combination Weighting Method:

To fully reflect the hierarchical structure of the PSR model and avoid the dominance of a single indicator with high variance, we adopted a hierarchical combination weighting strategy. This process involved three sub-steps:

1. Calculation of Local Entropy Weights within Subsystems:

First, we calculated the objective entropy weights independently for the indicators within each subsystem (Pressure, State, and Response). Let ${n}_{k}$be the number of indicators in the k-th subsystem. The entropy ${e}_{kj}$ and local weight ${w}_{kj}^{Entropy}$ for the j-th indicator in the k-th subsystem were calculated as:

$${p}_{kj}=\frac{{r}_{kj}}{\sum_{i=1}^{m}{r}_{kj}}$$

$${e}_{kj}=-\frac{1}{\mathrm{ln}m}\sum_{i=1}^{m}{p}_{kj}\mathrm{ln}\left({p}_{kj}\right)$$

$${w}_{kj}^{Entropy}=\frac{1-{e}_{kj}}{\sum_{j=1}^{{n}_{k}}\left(1-{e}_{kj}\right)}$$

This local calculation ensures that the internal structure of data distribution within each ecological dimension is preserved.

(2) Determination of Category Weights via AHP:

Second, the Analytic Hierarchy Process (AHP) was used to determine the subjective weights of the three criterion layers (${W}_{P}$, ${W}_{S}$, ${W}_{R}$). To ensure the objectivity and accuracy of the weights, we invited 10 experts to perform pairwise comparisons. The individual judgement matrices provided by the experts were aggregated into a single group judgment matrix using the geometric mean method to reduce individual bias. The final aggregated pairwise comparison matrix is presented in Table S3, and the Consistency Ratio (CR) was calculated to be 0.001, indicating satisfactory consistency.

(3) Derivation of Global Combined Weights:

Finally, the global composite weight (${W}_{j}$) for each indicator was obtained by multiplying the category weight (AHP) by the local indicator weight (Entropy):

$${W}_{j}={W}_{Categor{y}_{\_k}}^{AHP}\times{w}_{kj}^{Entropy}$$

The final weights are presented in Table 2:

**Table 2:** Weights.

Index layer	Weight
P1: Population density	0.0300
P2: Per capita GDP	0.0536
P3: Net Urban-Agrarian Dominance Index	0.1607
P4: Per 10,000 yuan of GDP Industrial wastewater discharge	0.0019
P5: Per 10,000 yuan of GDP Industrial sulfur dioxide emissions	0.0038
S1: Annual precipitation	0.1595
S2: Elevation	0.0184
S3: Biological richness	0.0606
S4: Annual evaporation	0.1904
S5: NDVI	0.0078
S6: Slope	0.0946
S7: Road network density index	0.0186
R1: Proportion of tertiary industry to GDP	0.1553
R2: Rate of returning farmland to forest and grass	0.0433
R3: Conversion rate of ecological land to nonecological land	0.0015

Calculation of ESEW Index:

$$\mathrm{E}\mathrm{S}\mathrm{E}{\mathrm{W}}_{\mathrm{i}}=\sum_{\mathrm{j}=1}^{\mathrm{n}}\left({\mathrm{r}}_{\mathrm{i}\mathrm{j}}\times{\mathrm{w}}_{\mathrm{j}}\right)$$

Warning situation classification standards:

This study classifies the ESEW standards for the basin into five levels, as outlined in Table 3 below:

**Table 3:** Classification standard of ESEW value.

ESEW index	Security level	Warning level
0.0 ≤ ESEW ≤ 0.2	I	Severe warning
0.2 < ESEW ≤ 0.4	II	Moderate warning
0.4 < ESEW ≤ 0.6	III	Light warning
0.6 < ESEW ≤ 0.8	IV	Generally safe
0.8 < ESEW ≤ 1	V	Very safe

#### Spatial autocorrelation analysis

Moran’s I is a key metric in spatial autocorrelation analysis, used to examine spatial correlations. Moran’s I is divided into two types: Global Moran’s I and Local Moran’s I. After variance standardization, Global Moran’s I ranges from − 1.0 to 1.0, allowing for the determination of whether the distribution of attribute values within the region shows clustering, dispersion, or randomness. In contrast, Local Moran’s I produces high and low values, indicating the spatial clustering of similar attribute values within the study units.

Global Moran’s I:

$$I=\frac{n\sum_{i=1}^{n}\sum_{j=1}^{n}{W}_{ij}\left({x}_{i}-\stackrel{-}{x}\right)\left({x}_{j}-\stackrel{-}{x}\right)}{\left(\sum_{i=1}^{n}\sum_{j=1}^{n}{W}_{ij}\right)\sum_{i=1}^{n}({x}_{i}-\stackrel{-}{x}{)}^{2}}$$

Local Moran’s I:

$$I_{i} = \frac{{x_{i} - \bar{x}}}{{S^{2} }}\mathop \sum \limits_{{j = 1,j \ne i}}^{n} W_{{ij}} \left( {x_{j} - \bar{x}} \right)$$

$${S}^{2}=\frac{1}{n}\sum_{i=1}^{n}({x}_{i}-\stackrel{-}{x}{)}^{2}$$

Where $\mathrm{n}$ represents the total number of spatial units in the study area, and ${\mathrm{W}}_{\mathrm{i}\mathrm{j}}$ denotes the weight matrix between each spatial unit $\mathrm{i}$ and $\mathrm{j}$ within the research scope. The ecological security evaluation scores at locations $\mathrm{i}$ and $\mathrm{j}$ are denoted as ${\mathrm{x}}_{\mathrm{i}}$ and ${\mathrm{x}}_{\mathrm{j}}$, respectively. The mean ecological security evaluation score across the study area is represented as $\stackrel{-}{\mathrm{x}}$.

#### Spatial econometric model

Model 1: Spatial Error Model (SEM)

The Spatial Error Model (SEM) is primarily used when spatial dependence is more evident in the error terms than in the dependent variable itself. By incorporating a spatial correlation structure into the random error terms, the SEM captures spatial autocorrelation caused by omitted variables, measurement errors, or other unobservable spatial factors. The general form of the SEM is expressed as follows:

$$Y=\alpha +\beta X+\theta ,\theta =\varepsilon W\theta +\sigma$$

Model 2: Spatial Lag Model (SLM)

The Spatial Lag Model (SLM) is primarily used to characterize the spatial interdependence of the dependent variable itself. It suggests that the observation in a given region is influenced by the levels of the same variable in neighboring regions. The specific form of the SLM is presented as follows:

$$Y=\alpha +\rho WY+\beta X+\theta$$

Model 3: Spatial Durbin Model (SDM)

The Spatial Durbin Model (SDM) is a generalization of both the Spatial Error Model (SEM) and the Spatial Lag Model (SLM). It is derived by reorganizing and expanding the SEM and SLM, adding additional conditional constraints to form the SDM. This model is used to analyze the interrelationships and spatial spillover effects among variables in spatial data. Unlike the SEM and SLM, the SDM not only accounts for the spatial autocorrelation of the dependent variable but also includes the spatial lag terms of the independent variables, thus capturing spatial dependencies more comprehensively. The general form of the SDM is expressed as follows:

$$Y=\alpha +\rho WY+\beta X+\lambda WX+\theta$$

Where $\mathrm{Y}$ is the dependent variable, $\mathrm{X}$represents the independent variables, ${\upalpha}$ is the constant term, $\mathrm{W}$ is the spatial weight matrix, ${\upbeta}$ is the vector of regression coefficients, ${\uprho}$ is the spatial lag coefficient for the dependent variable, indicating the degree of spatial autocorrelation, ${\uplambda}$ is the coefficient for the spatial lag of the independent variables, capturing the spatial spillover effects of these variables, ${\uptheta}$ is the error term, ${\upsigma}$ is the normally distributed independent and identically distributed white noise term, $\epsilon$ is the spatial error autoregressive coefficient to be estimated.

## Results and discussion

### Ecological security early warning analysis

**Fig. 2:**
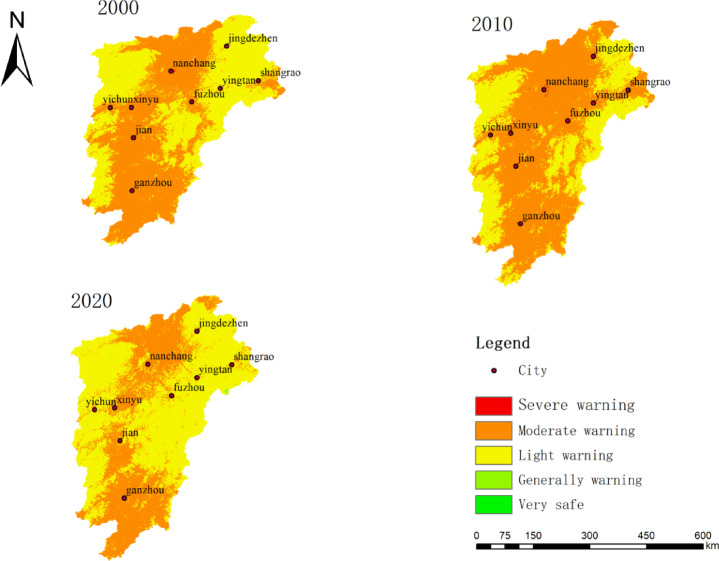
Changes in warning levels (The map was generated using ArcMap10.8 http://www.esri.com/).

The results indicate a clear two-stage evolution in early-warning intensity across the watershed. Areas classified as Moderate Warning first expanded and then contracted in the later period, whereas Light Warning areas showed the opposite pattern (an initial contraction followed by expansion). Spatially, Moderate Warning zones were concentrated mainly in the central–northern and southern parts of the basin, while Light Warning zones were more prevalent in the western and eastern areas, highlighting pronounced spatial disparities. Figure 2 summarizes the ESEW status for 2000, 2010, and 2020. In 2000, most of the basin fell into the Moderate and Light warning categories, with a relatively dispersed distribution. By 2010, the Moderate Warning extent expanded markedly, implying a basin-wide deterioration in ecological security. By 2020, the pattern partially reversed: Light Warning areas increased and Moderate Warning areas declined, and some locations shifted toward generally safe conditions, suggesting an overall improvement in ecological security in much of the basin. Notably, in both 2000 and 2010, a severe-warning core emerged in the north-central portion of the basin, closely associated with zones of intensive human activity, particularly around Nanchang City; this cluster weakened by 2020, consistent with a reduction in local ecological pressure. Despite the general improvement after 2010, areas classified as Generally Safe or Very Safe remained limited.

Pixel-based statistics (Table S2 in the Supplementary Materials) further confirm a substantial structural shift in ecological security. In 2000, the basin was almost evenly divided between Moderate Warning (Level II; 52.40%) and Light Warning (Level III; 47.54%). By 2010, the system shifted toward higher risk, with Moderate Warning peaking at 64.53% and Light Warning decreasing to 35.39%, consistent with heightened pressure during the decade of rapid industrialization. By 2020, a pronounced reversal occurred: Moderate Warning declined to 37.93%, while Light Warning became dominant at 62.00%. Categories at the extremes—Generally Safe and Severe Warning—remained spatially negligible (each < 0.1% of total area). Overall, the large-scale transition from Moderate to Light warning levels supports the interpretation of a basin-wide de-escalation of ecological pressure during 2010–2020, although persistent hotspots indicate that improvements were uneven and spatially concentrated.

While this study uses Biological Richness and NDVI as basin-scale proxies for ecological state, we recognize that the Poyang Lake wetland system is strongly shaped by seasonal hydrological fluctuations and biological migration dynamics. Recent research emphasizes that water-level seasonality is a key determinant of habitat suitability for migratory birds, including the Siberian crane. Due to constraints on consistent, county-comparable datasets, we did not explicitly include hydrological connectivity metrics or migration-corridor indicators as spatial covariates in the basin-scale SDM; nevertheless, their effects are partially reflected through the State-related indicators. For example, wetland vegetation degradation—captured by NDVI patterns and land-use conversion—often represents an ecological response to altered hydrological connectivity and human encroachment. Accordingly, the Moderate Warning status observed in the lake region likely reflects the cumulative outcomes of these interacting stressors. Future work that focuses on the lake–wetland interface would benefit from integrating high-frequency hydrological modeling and ecological movement data to complement the basin-scale early-warning assessment presented here.

**Fig. 3:**
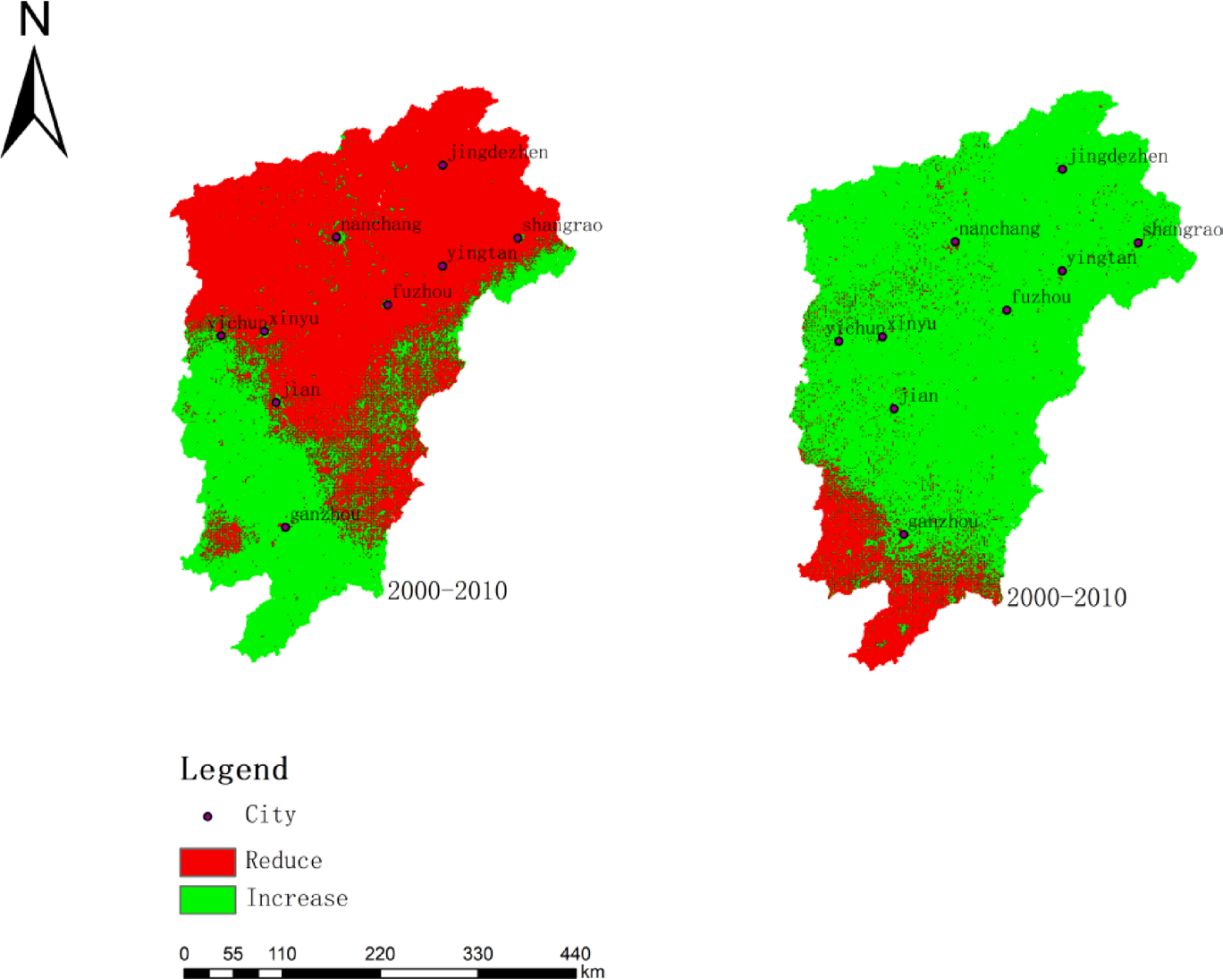
Changes in warning values (The map was generated using ArcMap10.8 http://www.esri.com/).

To further illustrate the changes in ESEW values within the basin, Fig. 3 shows the regions where these values increased or decreased during the periods of 2000–2010 and 2010–2020. As shown in the figure, from 2000 to 2010, the majority of the Poyang Lake Basin experienced a decline in ESEW values, indicating a deterioration of the local ecological environment during this period. In contrast, certain areas, such as the southwestern region, saw an increase in early-warning values, suggesting improvements in local ecological conditions. From 2010 to 2020, a larger number of regions showed rising early-warning values, reflecting an overall improvement in ecological conditions. However, the southern part of the basin still experienced some degree of ecological degradation. Notably, a few areas in the southwest saw continuous declines in early-warning values during both 2000–2010 and 2010–2020, underscoring the need for targeted monitoring and remediation efforts in these areas.

These findings highlight the spatial heterogeneity of ecological security trends within the Poyang Lake Basin over the past two decades. While overall progress has been made in improving ecological conditions, persistent challenges in certain regions require targeted environmental management strategies to ensure comprehensive and sustainable ecological security across the entire basin.

### Spatial correlation analysis of ecological security

Ecological conditions in neighboring regions are intricately connected to local ecosystems. Ecosystems exhibit connectivity and diffusion characteristics; therefore, changes in adjacent areas—such as variations in vegetation cover, water quality, and land use—can directly or indirectly affect the local ecological balance through hydrological cycles, atmospheric flows, and biological migration. Conversely, socioeconomic activities in neighboring regions, including agricultural practices and industrial emissions, as well as ecological management measures, significantly impact local conditions through resource flows and environmental diffusion. Consequently, investigating how the ecology of neighboring regions influences local ecosystems and identifying key external drivers are essential for understanding the dynamic relationships within the basin. This analysis provides a scientific basis for coordinated regional governance. Accordingly, this study shifts the analytical focus from the grid scale to the county scale to explore spatial interactions between each county and its neighbors.

#### Principal component analysis

Since the evaluation system comprises 15 indicators, multicollinearity is likely. Therefore, Principal Component Analysis (PCA) was performed to extract key components and reduce dimensionality. The suitability of the data was first assessed. As shown in Table 4, the Kaiser-Meyer-Olkin (KMO) measure was 0.793, exceeding the 0.6 threshold, and Bartlett’s test of sphericity was significant (*p* < 0.05), confirming the dataset’s suitability for PCA.

**Table 4:** KMO and Bartlett test results.

KMO and Bartlett test		
KMO		0.793
Bartlett test	Chi-square	5106.26
	df	105
	p-value	0.000

All indicators were standardized before analysis. Table 5 presents the extraction results and variance contributions. Four principal components with eigenvalues greater than 1 were selected, explaining 38.48, 18.10, 11.26, and 7.86% of the variance, respectively. The cumulative variance explained reached 75.70%. The linear combination coefficients for each component are also detailed in Table 5.

**Table 5:** Principal component analysis results.

Index	PcaScore1	PcaScore2	PcaScore3	PcaScore4
Eigenvalue	5.771	2.716	1.689	1.179
Variance Explained	38.48%	18.10%	11.26%	7.86%
Per capita GDP	0.2689	0.3081	0.2756	0.23
Per 10,000 yuan of GDP Industrial sulfur dioxide emissions	0.2703	0.1652	0.4634	0.2459
Per 10,000 yuan of GDP Industrial wastewater discharge	0.2909	0.1092	0.4429	0.2775
Net Urban-Agrarian Dominance Index	0.0884	0.4515	0.3123	0.1523
Conversion rate of ecological land to nonecological land	0.3202	0.1943	0.2339	0.1486
Annual evaporation	0.0851	0.4088	0.1685	0.4069
Population density	0.3502	0.137	0.0921	0.0037
Road network density index	0.3958	0.0299	0.0846	0.0802
Proportion of tertiary industry to GDP	0.3053	0.3075	0.2184	0.1618
Rate of returning farmland to forest and grass	0.0967	0.2068	0.103	0.6678
Annual precipitation	0.0963	0.2095	0.0323	0.1836
Biological richness	0.1953	0.0874	0.0939	0.1585
NDVI	0.3629	0.1742	0.0593	0.0671
Elevation	0.2134	0.3427	0.3519	0.1871
Slope	0.2143	0.3173	0.3484	0.1481

#### Spatial correlation test

Step 1: Global spatial autocorrelation test

This study first constructs an inverse distance spatial weight matrix, defined by the following formula:

$${w_{iz}}=\left\{ \begin{gathered} \frac{1}{{d_{{iz}}^{2}}},\,\,\,\,i \ne z \hfill \\ 0,\,\,\,\,\,\,\,\,i=z \hfill \\ \end{gathered} \right.$$

Where $\mathrm{i}\mathrm{a}\mathrm{n}\mathrm{d}\mathrm{z}$ represent counties. Specifically, the spatial weight between two counties is equal to the reciprocal of the square of the distance between their geometric centroids. The distance calculations were based on the projected coordinate system, and the resulting matrix was row-standardized to ensure meaningful interpretation of spatial spillovers. Unlike a 0–1 weight matrix, this approach assigns weights that decrease as the distance between counties increases. This weight matrix is utilized to perform a global spatial autocorrelation test, and the results are presented in Table 6 below:

**Table 6:** Global Moran’s I of watershed ESEW index.

Year	I	Z	*P*
2000	0.434	11.056	0.000
2010	0.461	11.750	0.000
2020	0.424	10.777	0.000

The results indicate that from 2000 to 2020, the global Moran’s I of the ESEW index in the Poyang Lake Basin was positive and significant, with p-values below 0.01, thus passing the 99% confidence level test. This demonstrates a strong spatial positive autocorrelation of the ESEW index in the Poyang Lake Basin, characterized by “high-high clustering” or “low-low clustering” patterns. In terms of the magnitude of the global Moran’s I, the ESEW index in the basin consistently exceeded 0.4 from 2000 to 2020, indicating a substantial level of spatial correlation.

Step 2: Local spatial autocorrelation test

**Fig. 4:**
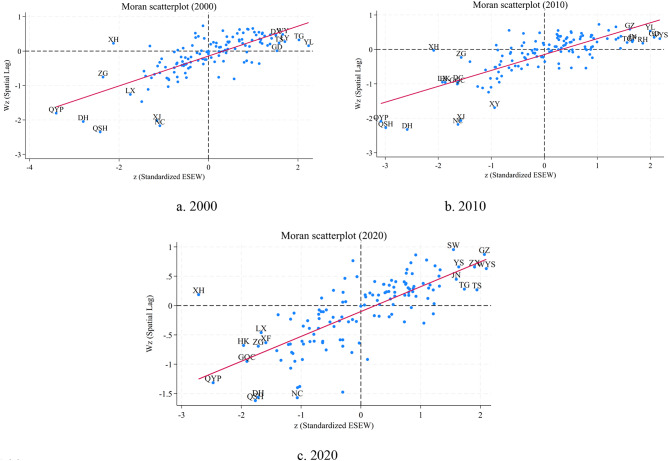
Moran scatter plot of watershed ESEW index.

Local spatial autocorrelation can illustrate the similarity of the ESEW index between a county and its neighboring counties, reflecting how spatial correlations change with location. In this study, Moran scatter plots were used to analyze local spatial autocorrelation. Using Stata software, Moran scatter plots for the ESEW index of each county were generated for the years 2000, 2010, and 2020. As shown in Fig. 4, the majority of counties’ ecological security early warning indices fall within the first and third quadrants, corresponding to “high-high clustering” and “low-low clustering,” respectively. This indicates a relatively significant spatial positive autocorrelation in the ESEW index within the basin.

As shown in the figure, counties such as Wuyuan, Wuyishan, Tonggu, and Yanling fall in the first quadrant, indicating high-value spatial clustering. These areas generally exhibit relatively favorable ecological conditions, as reflected by the well-recognized environmental quality of places such as Wuyuan. Nevertheless, high-value clusters should not be interpreted as risk-free; maintaining ecological advantages requires continued attention to emerging pressures and the design of targeted preventive policies.

In contrast, counties including Qingyunpu, Qingshan Lake, Donghu, Xunyang, Nanchang, and Lianxi are located in the third quadrant, forming a low-value clustering pattern concentrated around the provincial capital (Nanchang) and the urban core of Jiujiang. This configuration is plausibly associated with higher population density and greater pollutant emissions, which together generate stronger anthropogenic pressure and comparatively weaker ecological conditions than in surrounding counties.

Several counties appear as spatial outliers—most notably Yushui and Fengxin—suggesting local trajectories that deviate from the dominant neighborhood pattern. Yushui District, an important industrial base (e.g., steel and new energy industries), is shaped by policy-driven industrial restructuring. Its high development intensity contrasts with adjacent predominantly agricultural counties, producing localized heterogeneity in which elevated resource use and emissions pressures coexist with strengthened pollution-control interventions. Fengxin County, by comparison, lies at the ecotone between the Nanchang metropolitan area and the western mountainous ecological barrier. Its outlier status likely reflects a tension between strong natural endowments (mountain forests and associated ecosystem services) and adverse urbanization-related spillovers from the neighboring provincial capital, resulting in a more complex ecological security pattern than would be expected from regional clustering alone.

#### Variable selection and descriptive statistics

In this study, the ESEW index of the basin is selected as the dependent variable, while the four previously identified principal components are used as explanatory variables. Since the principal component scores derived from PCA are standardized variables centered at zero, they contain negative values, for which logarithmic transformation is mathematically undefined. To address this, we applied a Linear Shift Method (offsetting) before transformation. Specifically, a constant C was added to each principal component score to ensure all values are positive. The transformation formula is as follows:

$$L{n}_{P}caScor{e}_{ij}=\mathrm{l}\mathrm{n}(PcaScor{e}_{ij}+|\mathrm{m}\mathrm{i}\mathrm{n}\left(PcaScor{e}_{j}\right)|+1)$$

Where $PcaScor{e}_{ij}$ represents the original score of the j-th principal component for county i, and min (PcaScore_j) is the minimum value of that component across all samples. The addition of 1 ensures that the transformed values remain non-negative (since ln(1) = 0), preserving the relative order and variance structure of the data for the subsequent SDM analysis. All variables were subjected to logarithmic transformation, resulting in the following Table 7 of variable indices for the influencing factors.

**Table 7:** Variables of factors affecting ESEW in the basin.

Types	Variable
Explained variable	Ln_esew
Explanatory variables	Ln_PcaScore1
	Ln_PcaScore2
	Ln_PcaScore3
	Ln_PcaScore4

Table 8 presents the descriptive statistical results for all variables. Since the original data for the ESEW index in the basin range between 0 and 1, their logarithmic transformations are all negative. After applying logarithmic transformations to all variables, the standard deviations are relatively small, indicating that the data exhibit low volatility.

**Table 8:** Descriptive statistics of variables.

Variable	Obs	Mean	Std. dev.	Min	Max
Ln_esew	396	-0.92	0.13	-1.41	-0.59
Ln_PcaScore1	396	0.80	0.68	-4.60	3.03
Ln_PcaScore2	396	1.16	0.59	-4.60	2.76
Ln_PcaScore3	396	1.90	0.37	-4.60	2.75
Ln_PcaScore4	396	1.65	0.36	-4.60	2.49

#### Model selection

To investigate the spatial relationships among the ecological security statuses within the basin and determine which factors from neighboring counties influence a county’s ecological security, three spatial econometric models were constructed in the preceding sections. Following the methodology of Anselin L^35^, the selection among these three models is carried out based on the following steps, and the most appropriate model is used for regression analysis.

Step 1: LM and Robust-LM tests

The LM-Error and Robust-LM-Error tests are used to detect the presence of spatial correlation in the error terms (i.e., to assess the need for a spatial error model). If the LM-Error or Robust-LM-Error statistics are significant, it indicates that the residuals of the simple linear model exhibit spatial dependence, which necessitates incorporating the spatial dependence structure of the error terms into the model. The LM-Lag and Robust-LM-Lag tests are used to determine whether the dependent variable itself exhibits spatial lag effects (i.e., to assess the need for a spatial lag model). If the LM-Lag or Robust-LM-Lag statistics are significant, it implies that, in addition to the explanatory variables, the spatially lagged values of the dependent variable also significantly impact the explanatory variables of the current region.

As shown in Table 9 below, both the LM and Robust-LM tests reject the null hypothesis for the error terms and lag terms at highly significant levels (with p-values approaching 0). This indicates the presence of strong spatial correlation characteristics in the data. Simple linear models are insufficient for capturing these spatial features effectively, and therefore, a Spatial Durbin Model (SDM) should be used to better explain the spatial correlations within the data.

**Table 9:** LM and Robust-LM test results.

Test	Statistic	p-value
LM-Error	290.886	0.000
Robust-LM-Error	87.762	0.000
LM-Lag	213.294	0.000
Robust-LM-Lag	10.169	0.001

Step 2: Wald and LR tests

The Wald test and the LR test are used to determine whether the Spatial Durbin Model (SDM) degenerates. If the statistical results of both tests are significant, it indicates that the Spatial Durbin Model should be selected. Table 10 presents the test results, showing that the p-values for all test statistics are below 0.05, thus passing the 95% confidence level test. This result suggests that, under the traditional model specification without spatial terms, significant spatial correlations and transmission effects exist both in the error structure and in the dependent variable itself. Therefore, this study selects the Spatial Durbin Model (SDM) to better capture the pronounced spatial correlation characteristics in the data.

**Table 10:** Wald and LR test results.

Test	Statistic	p-value
Wald-Error	29.59	0.000
Wald-Lag	11.74	0.019
LR-Error	27.49	0.000
LR-Lag	13.05	0.011

#### Analysis of SDM model regression results

This study uses a fixed effects model for regression analysis. Fixed effects are further categorized into time fixed effects, individual fixed effects, and dual fixed effects. To select the most appropriate model, a Likelihood Ratio (LR) test is conducted to compare these three models. The results of the test are presented in Table 11 below:

**Table 11:** LR test results.

Likelihood-ratio test	Statistic	p-value
LR test for spatial effect	24.14	0.000
LR test for time effect	794.83	0.000

From the table above, it can be seen that all p-values are below 0.01, indicating that the dual fixed effects model is the optimal choice. Therefore, the dual fixed effects Spatial Durbin Model (SDM) is selected, and the constructed SDM model is presented as follows:

$$\begin{aligned} Ln\_ese{w_{it}} & =\alpha +\rho WLn\_ese{w_{zt}}+{\beta _1}Ln\_PcaScore{1_{it}} \\ & +{\beta _2}Ln\_PcaScore{2_{it}}+{\beta _3}Ln\_PcaScore{3_{it}} \\ & +{\beta _4}Ln\_PcaScore{4_{it}}+{\lambda _1}WLn\_PcaScore{1_{zt}} \\ & +{\lambda _2}WLn\_PcaScore{2_{zt}}+{\lambda _3}WLn\_PcaScore{3_{zt}} \\ & +{\lambda _4}WLn\_PcaScore{4_{zt}}+{\theta _{it}} \\ \end{aligned}$$

Where i and z represent different counties, $\mathrm{t}$ denotes the year. Using this model for regression analysis, the results are presented in Table 12 below:

**Table 12:** Regression results of the SDM model.

Variable	SDM
Ln_PcaScore1	0.0173***(2.99)
Ln_PcaScore2	-0.0013(-0.28)
Ln_PcaScore3	-0.128***(-5.49)
Ln_PcaScore4	0.132***(5.64)
W*Ln_PcaScore1	-0.0408* (-1.76)
W*Ln_PcaScore2	0.00169(0.15)
W*Ln_PcaScore3	-0.191***(-2.86)
W*Ln_PcaScore4	0.171***(2.66)
rho	0.828***(19.53)
sigma^2^_e	0.000578***(13.57)
AIC	-1770.115
BIC	-1730.301
N	396

t statistics in parentheses, *, ** and *** represent respectively significance levels at the 10, 5 and 1%.

The results clearly show that after controlling for dual fixed effects, the spatial lag effects of the third and fourth principal components are both significant. This indicates that factors from neighboring counties have a notable impact on the ESEW index of the current county. Additionally, the spatial lag coefficient of the dependent variable is 0.828 and highly significant, suggesting a strong positive spatial autocorrelation of the dependent variable. This means that the ecological security status of neighboring counties significantly influences the ecological security status of the current county. The spatial Durbin Model provides a dynamic early-warning function by capturing how ecological pressures originating in adjacent counties propagate across space and time. The strong spatial autoregressive coefficient (rho = 0.828) reveals that ecological risks in the basin exhibit high persistence and fast transmission, meaning that deterioration in an upstream or neighboring unit can serve as an early-warning signal for subsequent degradation in downstream areas. This dynamic, path-dependent mechanism is essential under the “high dynamics” and “high complexity” emphasized in early-warning theory. Overall, the model exhibits lower AIC and BIC values, as well as a smaller sigma2_e, indicating a good fit and robust regression results.

#### Analysis of spatial effect decomposition results

Since the Spatial Durbin Model (SDM) includes lagged terms for each variable, it does not fully capture the interpretive effects of the regression coefficients. Therefore, further decomposition of the influencing factor variables is necessary. In this study, spatial effect decomposition is performed using Stata software, following the methodology of LeSage J^36^. The total effects of the explanatory variables are decomposed into direct and indirect effects, with the specific regression results presented in Table 13. The direct effects represent the impact of local explanatory variables on the local dependent variables, while the indirect effects reflect the influence of explanatory variables in neighboring areas on the dependent variables of the current region.

**Table 13:** Results of spatial effect decomposition.

Variable	LR_Direct	LR_Indirect	LR_Total
Ln_PcaScore1	0.0125(0.0084)	-0.149(0.156)	-0.136(0.162)
Ln_PcaScore2	-0.00135(0.005)	0.00573(0.0734)	0.00437(0.0767)
Ln_PcaScore3	-0.185***(0.026)	-1.809***(0.69)	-1.995***(0.703)
Ln_PcaScore4	0.186***(0.025)	1.722**(0.686)	1.908***(0.698)

Standard errors in parentheses, *, ** and *** represent respectively significance levels at the 10, 5 and 1%.

The PCA loadings (Table 5) indicate that Component 3 is primarily driven by industrial SO₂ emissions per CNY 10,000 of GDP, industrial wastewater discharge per CNY 10,000 of GDP, and the Net Urban–Agrarian Dominance Index (NUADI), with loading coefficients of 0.463, 0.443, and 0.312, respectively. Because the SDM spatial effect decomposition for Component 3 shows a significant indirect effect of − 1.809, the implied spillover contributions of these drivers from neighboring counties to the focal county’s ecological security are − 0.8376, − 0.8014, and − 0.5644, respectively (i.e., loading × indirect-effect coefficient). These results provide empirically grounded support for the early-warning relevance of the SDM: the model identifies which pressure variables are most strongly associated with cross-county deterioration, thereby enabling sensitivity-oriented diagnosis. In the log specification used here, the negative spillover associated with industrial SO₂ implies that a 1% increase in upstream (neighboring) industrial SO₂ emissions is associated with an approximate 0.84% decrease in ecological security in downstream or adjacent counties (based on the indirect-effect magnitude). Similarly, the significant negative spillover linked to NUADI suggests that urbanization pressure around the lake’s periphery is approaching a critical intensity. Without effective intervention, areas currently classified as Light Warning in the basin’s transition belt may be more likely to shift toward Moderate Warning as construction-land expansion and associated pressures accumulate. Substantively, these findings are consistent with the physical and socioeconomic connectivity of the basin: pollution emissions and high-intensity development in one county can propagate through air and water pathways and through interlinked production and settlement systems, thereby increasing ecological vulnerability beyond administrative boundaries.

Component 4 is chiefly associated with annual evaporation and the rate of returning farmland to forest and grass, with loadings of − 0.407 and 0.668, respectively. Given that the SDM decomposition for Component 4 yields a significant indirect effect of 1.722 (*p* < 0.05), the corresponding spillover contributions from neighboring counties are − 0.70085 for annual evaporation and 1.1502 for the rate of returning farmland to forest and grass. The negative spillover associated with evaporation indicates that unusually high evaporative demand in surrounding areas may intensify regional water–energy imbalance and increase ecosystem sensitivity in adjacent counties. In contrast, the positive spillover for returning farmland to forest and grass suggests that restoration actions implemented in nearby jurisdictions can generate measurable positive externalities, improving ecological security beyond the intervention boundary.

The spatial spillovers identified by the SDM also align with interpretable geographical mechanisms behind the observed clustering patterns. In low–low clusters (e.g., the Nanchang metropolitan area), negative spillovers from industrial pollution (SO₂ and wastewater) are plausibly amplified by the basin’s relatively flat terrain, dense hydrological network, and the mobility of environmental media: atmospheric transport can spread pollutants across borders, and river networks can transmit waterborne pollution downstream, producing a cross-border pollution coupling effect. In addition, metropolitan economic integration tends to create contiguous zones of high human disturbance, where urban expansion and infrastructure development in one county intensify land conversion pressures in adjacent counties, jointly weakening regional ecological security. By contrast, in high–high clusters (e.g., peripheral mountainous areas), positive spillovers from restoration are consistent with the continuity of natural geographic units. Continuous mountain systems and forest belts function as ecological corridors that cross administrative boundaries, and ecosystem services such as soil retention, water purification, and biodiversity support can generate positive externalities for neighboring downstream counties. This supports the interpretation that high–high clustering reflects underlying physical connectivity rather than a purely statistical artifact.

Because principal components are not directly interpretable as elasticities of the original variables, we further conducted a confirmatory SDM using representative raw indicators to validate the PCA-based inference. We selected industrial SO₂ emissions (pollution pressure), NUADI (urbanization pressure), rate of returning farmland to forest (response), and annual evaporation (state) for the confirmatory regression. The results (Table S1 in the Supplementary Materials) show that the directions of spillover effects are highly consistent with the PCA-based findings: industrial SO₂ and NUADI exhibit significant negative indirect effects (*p* < 0.05), confirming that pollution emissions and high-intensity urbanization in neighboring areas materially deteriorate local ecological security. This consistency reinforces the credibility of the mechanism interpretation derived from the spatial effect decomposition.

#### Robustness check

To ensure the robustness of the empirical results, this study conducts a robustness check by replacing the spatial weight matrix. Specifically, regression analysis is performed by substituting the inverse distance matrix with the Queen contiguity matrix (where two counties are considered adjacent if they share a boundary or even a single point) in the spatial adjacency matrix. The results are presented in Table 14:

**Table 14:** Regression results based on the Queen contiguity matrix.

Variable	Spatial effect of dependent variable	LR_Direct	LR_Indirect	LR_Total
Ln_PcaScore1		0.0126*(0.007)	-0.0796(0.058)	-0.0671(0.064)
Ln_PcaScore2		0.00075(0.005)	0.150***(0.049)	0.151***(0.052)
Ln_PcaScore3		-0.115***(0.023)	-0.389**(0.192)	-0.504**(0.206)
Ln_PcaScore4		0.137***(0.022)	0.326*(0.182)	0.462**(0.196)
rho	0.830***(0.028)			

Standard errors in parentheses, *, ** and *** represent respectively significance levels at the 10, 5 and 1%.

By comparing the results, it is clear that the direction and significance levels of the direct effects, indirect effects, and total effects for each explanatory variable have not changed significantly. Although the significance of some variables has shifted, with some coefficients increasing and others decreasing, the direction of these coefficients remains consistent. Additionally, the spatial effect of the dependent variable, the influence of neighboring counties’ ecological security levels on the current county—has fluctuated only slightly from 0.828 to 0.830, with no change in significance. Overall, the results obtained after replacing the matrix are essentially the same as those before the replacement, indicating that the findings of this study are robust when considering different spatial matrices.

As an additional robustness check, we re-estimate the SDM by replacing the inverse-distance and contiguity matrices with a water-network-based spatial weight matrix. In this specification, two counties are treated as neighbors if they are traversed by the same river segment, so that spatial interactions are transmitted along the hydrological network rather than along straight-line geometric distance. The long-run direct, indirect and total effects obtained under this water-system matrix are reported in Table 15.

**Table 15:** Regression results based on the water-network-based spatial weight matrix.

Variable	Spatial effect of dependent variable	LR_Direct	LR_Indirect	LR_Total
Ln_PcaScore1		-0.003*(0.01)	-0.182**(0.087)	-0.186*(0.095)
Ln_PcaScore2		0.009(0.006)	0.04(0.033)	0.049(0.036)
Ln_PcaScore3		-0.181***(0.031)	-0.193**(0.095)	-0.374***(0.112)
Ln_PcaScore4		0.187***(0.031)	0.125(0.083)	0.312***(0.099)
rho	0.625***(0.059)			

Standard errors in parentheses, *, ** and *** represent respectively significance levels at the 10, 5 and 1%.

Overall, the qualitative patterns remain highly consistent with the baseline specification. The first principal component (ln_PcaScore1) continues to exhibit a small negative association with ecological security: its long-run direct effect is modest in magnitude (–0.003, significant at the 10% level), whereas the long-run indirect effect is substantially larger (–0.182, significant at the 5% level). This implies that increases in this factor within a county are associated with a measurable deterioration of ecological security in hydrologically connected counties. The second principal component (ln_PcaScore2) shows no robust relationship with ecological security; both its direct and indirect effects remain small and statistically insignificant. In contrast, the third principal component (ln_PcaScore3), which primarily reflects industrial pollution pressure, remains strongly negative. Under the river-network-based weight matrix, the long-run direct effect is − 0.181 (significant at the 1% level) and the long-run indirect effect is − 0.193 (significant at the 5% level), yielding a large and highly significant total effect of − 0.374. This result reinforces the interpretation that industrial pollution not only undermines local ecological security but also propagates along river-connected pathways to affect downstream and adjacent counties. The fourth principal component (ln_PcaScore4), capturing ecological restoration and improvement, remains strongly positive: the long-run direct effect is 0.187 and the total effect reaches 0.312 (both significant at the 1% level), indicating that restoration efforts enhance ecological security both locally and through positive spillovers transmitted via the river network.

The spatial autoregressive coefficient of the dependent variable also remains large and highly significant (ρ = 0.625, *p* < 0.01), confirming strong spatial dependence in ecological security when spatial structure is defined by hydrological connectivity. Taken together, these robustness checks indicate that the study’s core conclusions—spatial clustering in ecological security and the spillover roles of industrial pollution and ecological restoration—are not sensitive to the choice of the spatial weight matrix and continue to hold when a water-system-based matrix is applied.

Relative to prior work that has emphasized static assessments or single-factor relationships in the Poyang Lake region, this study advances watershed ecological security research by shifting the analytical lens from isolated territorial evaluation to cross-jurisdictional spatial interaction. Whereas much of the existing literature implicitly treats administrative units as independent, the SDM framework used here quantifies spillover effects consistent with an upstream–midstream–downstream transmission mechanism. This contribution highlights the often underappreciated role of inter-county (and, where relevant, inter-provincial) interactions in shaping local ecological security, and it provides a stronger analytical basis for moving from fragmented management toward a basin-wide, collaborative early-warning approach.

## Conclusion

### Key findings on spatiotemporal patterns and cross-regional spillovers of ESEW

Using the Poyang Lake Basin as a case study, we developed a PSR-based ESEW framework at the 1-km grid scale and integrated it with county-level spatial analysis. We first characterized the spatiotemporal evolution of ESEW, then examined local spatial clustering, and finally employed the Spatial Durbin Model (SDM) to quantify cross-county spillovers.

From 2000 to 2020, ESEW levels across the basin remained generally low, indicating persistent ecological fragility. Spatially, higher warning intensities were consistently observed in the central–northern and southern parts of the basin, reflecting sustained and concentrated ecological stress. Over time, the proportion of grids classified as “Moderate Warning” declined slightly, while a small number of “Generally Safe” grids emerged, implying marginal improvements in specific locations. However, the overall ecological status remained precarious. This pattern aligns with the rapid industrialization and urbanization in the central–northern and southern subregions, where population concentration and land-use intensification generated compounded pressures, such as agricultural non-point source pollution and domestic sewage discharge. In response, priority areas should be targeted for strengthened monitoring, stricter pollutant discharge controls, and ecological restoration. Interventions such as riparian reforestation, wetland rehabilitation, and soil stabilization are particularly relevant for enhancing resilience and expanding “Generally Safe” areas.

Local spatial autocorrelation analysis revealed pronounced clustering. A “low–low” cluster centered on the Nanchang metropolitan area indicated contiguous counties sharing poor ecological conditions, likely reflecting common pressures from industrial emissions and urban expansion. By contrast, a “high–high” cluster around Wuyuan identified a spatially coherent zone with better ecological conditions, supported by favorable natural endowments and sustained protection. These patterns underscore a key implication: ecological risks and benefits are not confined by administrative borders. Degradation can diffuse across jurisdictions, just as improvements can generate positive externalities. Consequently, governance responses must be designed at the basin scale rather than as isolated county-level actions. Establishing formal cross-regional cooperation mechanisms is therefore essential, including unified monitoring protocols, joint satellite and field data sharing, and coordinated emergency response arrangements. A shared funding arrangement and incentive-compatible schemes, such as ecological compensation to upstream or better-performing jurisdictions, would further align local interests with basin-wide ecological security objectives.

The SDM results provide direct evidence that local ecological security is significantly shaped by neighboring conditions. The positive global spatial dependence indicates that improvements or deterioration in adjacent areas can transmit to local ecological outcomes, reinforcing the importance of maintaining continuous ecological networks and functional corridors. Effect decomposition highlights that higher industrial SO₂ emissions per CNY 10,000 of GDP and higher industrial wastewater discharge per CNY 10,000 of GDP in neighboring counties significantly erode local ecological security. Similarly, higher anthropogenic interference (reflecting land-use conversion, infrastructure expansion, and deforestation) and increased annual evaporation in neighboring regions exert negative spillovers, intensifying stress on water resources and marginal habitats. In contrast, a higher rate of returning farmland to forest and grassland in neighboring counties generates a robust positive spillover, indicating that restoration efforts in upstream or adjacent jurisdictions can measurably improve regional ecological conditions. Taken together, these findings support the necessity of coordinated governance that jointly targets pollution sources and land-use pressures while scaling up ecological restoration actions.

### Differentiated policy implications and operational implementation

Based on the spillover structure identified by the SDM, this study proposes an implementation pathway that emphasizes differentiated zoning control, cross-regional collaborative governance, and incentive-compatible ecological compensation.

First, a differentiated zoning-and-grading strategy should be adopted to address basin heterogeneity. In low–low agglomeration areas (e.g., the Nanchang metropolitan area), the priority should be source control through a clear negative list for industrial access. Given the strong negative spillovers of industrial SO₂ and wastewater, local governments should strictly restrict additional high-emission projects, particularly in chemical and heavy manufacturing sectors. Operationally, a grid-informed monitoring network should be deployed near administrative boundaries, supported by automated continuous emission monitoring systems where feasible, to detect cross-border pollutant transmission in a timely manner. In high–high agglomeration areas (e.g., Wuyuan and Wuyishan), policy should shift from protection-only approaches toward ecological valorization and corridor conservation. Because these areas generate positive externalities through restoration and ecosystem service provision, piloting Gross Ecosystem Product (GEP) accounting can help quantify ecological value and support the development of eco-branding strategies (e.g., a basin-wide “Poyang Lake Green Label”) that convert ecological advantages into sustainable economic returns.

Second, cross-regional joint prevention should be institutionalized through a River Chief joint coordination mechanism. The results indicate that administrative fragmentation can amplify pollution spillovers; accordingly, a basin-level coordination platform should be established to align emission standards across jurisdictions and prevent “pollution haven” dynamics in which high-emission industries relocate to counties with weaker regulation. In addition, an integrated data platform that combines meteorological, hydrological, and pollution information is necessary to support synchronized early warning and joint emergency response.

Third, ecological compensation should evolve from ad hoc, government-led transfers toward more incentive-compatible and market-oriented arrangements. Because upstream restoration often generates downstream benefits, a diversified compensation scheme is required. Vertical compensation can be supported through a dedicated provincial restoration fund that prioritizes transfer payments to upstream counties that bear development constraints for conservation. Horizontal compensation can be operationalized through a performance-based cross-section water-quality contract, under which downstream beneficiary cities compensate upstream protector counties when water-quality targets are met, while penalties apply when targets are not achieved. Such arrangements directly tie fiscal incentives to ecological outcomes and improve enforcement credibility.

To institutionalize upstream–downstream benefit sharing, a coupled benefit-sharing mechanism is further recommended. A bilateral, contract-based “ecological performance agreement” can be implemented using cross-section water-quality monitoring: when upstream jurisdictions deliver water quality exceeding the agreed threshold, downstream jurisdictions pay an ecological service purchase fee; when performance falls below the baseline, upstream jurisdictions pay a pollution compensation fee. In parallel, an opportunity-cost-based lateral transfer scheme should be introduced so that compensation reflects not only direct restoration costs but also foregone development opportunities under industrial restrictions. An “enclave economy” approach can be considered, in which upstream governments obtain land-use or tax-sharing rights in downstream industrial parks, allowing upstream areas to share in the economic dividends of downstream industrial activity while maintaining conservation incentives.

Finally, to address high evaporation-related stress, an integrated landscape management approach is needed. Agricultural agencies should subsidize water-saving irrigation technologies to replace flood irrigation. Meanwhile, legally mandated riparian buffer zones (e.g., 50–100 m along main channels, subject to local feasibility and existing land tenure) should be established to intercept agricultural non-point source pollution before it enters the lake and river system.

### Methodological robustness and sensitivity analysis

**Fig. 5:**
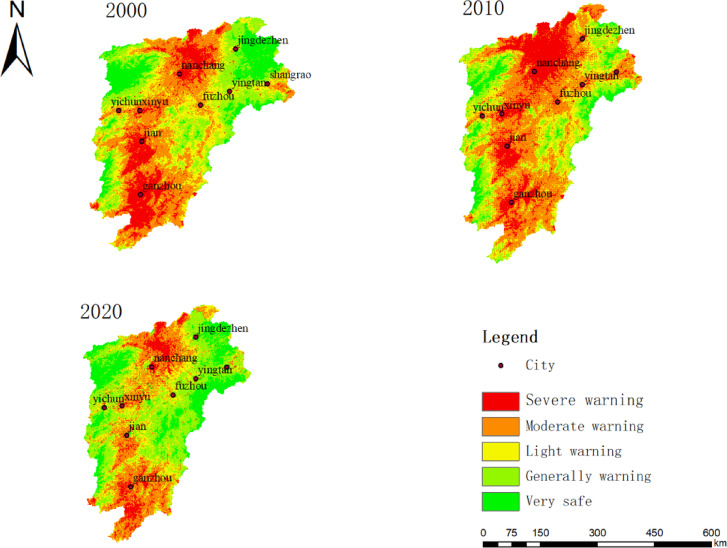
Changes in warning levels (Jenks) (The map was generated using ArcMap10.8 http://www.esri.com/).

To mitigate concerns about arbitrariness in threshold selection, we conducted a sensitivity analysis comparing the baseline Equal Interval classification (0.2 increments) with the Natural Breaks (Jenks) method. As shown in Fig. 5, the spatial distribution of ecological security classes is highly consistent across the two approaches. The Jenks method, which minimizes within-class variance, identifies Severe Warning and Moderate Warning hotspots in the same key locations as the Equal Interval method, particularly in the Nanchang metropolitan area and the southern industrial clusters. The spatial overlap of high-risk zones exceeds 90%, indicating that the main spatial patterns are robust and not artifacts driven by a specific classification rule.

Furthermore, to ensure the robustness of the weighting scheme, we compared the baseline hierarchical combination weighting (AHP-Entropy) with an Entropy-only weighting approach. A Spearman’s rank correlation analysis was performed on the county-level ESEW indices for each period. The results revealed a consistently strong positive correlation between the two methods, with Spearman’s rho values of 0.898 in 2000, 0.824 in 2010, and 0.850 in 2020 (all *p* < 0.001). This indicates that while the specific weighting method causes minor fluctuations in absolute values, the relative ranking and spatial identification of high-risk versus safe zones remain highly stable. Therefore, the main conclusions regarding the basin’s spatiotemporal evolution are not sensitive to the specific choice of weighting parameters.

## Supplementary Information

Below is the link to the electronic supplementary material.

Supplementary Material 1

## Data Availability

The raw datasets analyzed in this study are publicly available from the following repositories: (1) Land use, GDP, population, and NDVI data are available at the Resource and Environmental Science Data Platform (http://www.resdc.cn/); (2) Meteorological data are available from the China Meteorological Data Service Centre (http://data.cma.cn/); (3) Elevation data are available from the Geospatial Data Cloud (https://www.gscloud.cn/). The processed county-level panel data and the Stata code used for the SDM analysis are available from the corresponding author upon reasonable request.
