# Supplementary material for: Spatial early-warning assessment of ecological security in the Poyang Lake Basin based on PSR and Spatial Durbin Modeling
Source: Sci Rep. 2026 Apr 24;16:18967. doi: 10.1038/s41598-026-44479-4 (PMC13275731; doi:10.1038/s41598-026-44479-4)
Supplement: Supplementary file 1 — Supplementary Material 1 [file 41598_2026_44479_MOESM1_ESM.docx]

**Appendix**

Table S1

| Variable | Spatial effect of dependent variable | LR_Direct | LR_Indirect | LR_Total |
| --- | --- | --- | --- | --- |
| ln_Annual evaporation |  | -0.86***(0.068) | -0.92(0.68) | -1.78***(0.67) |
| ln_ rate of returning farmland to forest |  | 0.013***(0.004) | 0.054(0.069) | 0.067(0.071) |
| ln_so2 |  | -0.038(0.026) | -0.801**(0.33) | -0.839**(0.349) |
| ln_ NUADI |  | -0.087(0.056) | -1.97**(0.98) | -2.065**(1.015) |
| rho | 0.824***(0.042) |  |  |  |

Notes: Standard errors in parentheses, *, ** and *** represent respectively significance levels at the 10%, 5% and 1%.

Table S2

| Year | Security Level | Pixel Count | Area Proportion (%) |
| --- | --- | --- | --- |
| 2000 | Severe warning | 51 | 0.03% |
|  | Moderate warning | 84,297 | 52.40% |
|  | Light warning | 76,477 | 47.54% |
|  | Generally safe | 56 | 0.03% |
|  | Very safe | 0 | 0.00% |
| 2010 | Severe warning | 79 | 0.05% |
|  | Moderate warning | 103,765 | 64.53% |
|  | Light warning | 56,908 | 35.39% |
|  | Generally safe | 43 | 0.03% |
|  | Very safe | 0 | 0.00% |
| 2020 | Severe warning | 1 | 0.00% |
|  | Moderate warning | 61,096 | 37.93% |
|  | Light warning | 99,863 | 62.00% |
|  | Generally safe | 103 | 0.06% |
|  | Very safe | 0 | 0.00% |

Table S3 The aggregated pairwise comparison matrix of the criterion layer (PSR).

|  | P | S | R |
| --- | --- | --- | --- |
| P | 1 | 0.455 | 1.25 |
| S | 2.2 | 1.00 | 2.75 |
| R | 0.8 | 0.364 | 1 |

Note: The matrix was constructed by aggregating the scores of 10 experts. The consistency ratio (CR) is 0.001, which is less than 0.1, indicating satisfactory consistency.
